# Epidemic Contact Tracing via Communication Traces

**DOI:** 10.1371/journal.pone.0095133

**Published:** 2014-05-01

**Authors:** Katayoun Farrahi, Rémi Emonet, Manuel Cebrian

**Affiliations:** 1 Department of Computing, Goldsmiths, University of London, London, United Kingdom; 2 Department of Machine Learning, Laboratoire Hubert Curien, Saint-Etienne, France; 3 Media Laboratory, Massachusetts Institute of Technology, Cambridge, Massachusetts, United States of America; 4 Department of Computer Science and Engineering, University of California at San Diego, La Jolla, California, United States of America; 5 National Information and Communications Technology Australia, Melbourne, Victoria, Australia; University of Namur, Belgium

## Abstract

Traditional contact tracing relies on knowledge of the interpersonal network of physical interactions, where contagious outbreaks propagate. However, due to privacy constraints and noisy data assimilation, this network is generally difficult to reconstruct accurately. Communication traces obtained by mobile phones are known to be good proxies for the physical interaction network, and they may provide a valuable tool for contact tracing. Motivated by this assumption, we propose a model for contact tracing, where an infection is spreading in the physical interpersonal network, which can never be fully recovered; and contact tracing is occurring in a communication network which acts as a proxy for the first. We apply this dual model to a dataset covering 72 students over a 9 month period, for which both the physical interactions as well as the mobile communication traces are known. Our results suggest that a wide range of contact tracing strategies may significantly reduce the final size of the epidemic, by mainly affecting its peak of incidence. However, we find that for low overlap between the face-to-face and communication interaction network, contact tracing is only efficient at the beginning of the outbreak, due to rapidly increasing costs as the epidemic evolves. Overall, contact tracing via mobile phone communication traces may be a viable option to arrest contagious outbreaks.

## Introduction

There is great potential to deepen our understanding of disease dynamics through the analysis of digital traces of individual and collective behaviour [1]–[7]. This is because, traditionally, the collection of how epidemics propagate in a population has been based on individual self-reporting, known to be severely biased [8]–[10]. The bias is documented both at the individual level, self-reporting of symptoms, but also about the recollection of recent face-to-face interactions that potentially lead to contagion [11]. Increasing awareness of personal data privacy are unlikely to lighten this limitation in the near future [12].

We already have some examples in the digital epidemiology direction which use large-scale digital traces for simulation. For instance, a large-scale sociotechnological network based on Facebook data was used to study the role of community structure in disease dynamics [13]. Also, close proximity interactions (CPIs) captured using wireless sensors were able to map the fine-grained, face-to-face interactions of a community [14]. By studying the CPIs on an American high school community and recovering the contact network, new prevention strategies were designed with the potential to be more effective than random immunization. Infectious disease dynamics have been simulated focusing on the temporal and heterogeneity aspects considering CPIs sensed over a two day period at a conference using RFID tags [15].

While these previously investigated sources of digital sensing (Facebook and CPIs from wearable badges) are advantageous in that they capture large scale interactions in a continuous manner giving a more complete estimate of human interactions in reality, they also present some limitations. Online social networks represent online social behaviours which differ from physical proximity interactions whereby disease transmission occurs and may fail to capture the fine-grained, face-to-face interaction dynamics relevant for disease transmission [10]. CPI monitoring using wearable badges is a costly and limited resource requiring participants to wear an additional sensing device and therefore cannot readily be extended to the population at large; CPIs may not be readily extended to larger scales in the immediate future (e.g. Salathé *et al.* consider one school day [14], Stehlé *et al.* consider two conference days [15], and Isella *et al.* consider one week [16]).

In this regard, mobile phones provide a promising resource as they are ubiquitously carried by the population, irrespectively of socio-economic status, and provide a much larger-scale, data-driven opportunity for epidemiology. Further, mobile phones are carried by people when they travel overseas, potentially serving as a global physical proximity sensor. Its pervasiveness in countries under development, where pandemic prevention is most critical, makes then a viable option [17].

Our present effort focuses on exploiting these phone communication and interaction traces for epidemic simulation and contact tracing [18]. Communication traces obtained by mobile phones are known to be good proxies for the physical interaction network [19]–[21], and therefore our goal is to consider how to simulate a contact tracing model over mobile sensed interaction data. We aim at exploring the potential of communication datasets to serve as a realistic cue for physical proximity interactions at large.

We develop a model where the infection takes place over the close-proximity physical network (which can never be fully recovered in reality), and assume contact tracing occurs on a differing network, in this case a communication (phone, sms) inferred one. We explore the contact tracing model proposed in detail, particularly focusing on tracing efforts on noisy networks, representing a perturbed subset of the ideal network. Finally, we simulate our proposed model over the real mobile phone interaction data dynamics, demonstrating mobile phone interactions are a promising tool for large-scale epidemic simulations, and mobile phone communication logs can be used as a concrete source for contact tracing reducing the effects of an epidemic. Just as optimizing immunization strategies is of great interest if only incomplete immunization is possible [14], optimizing contact tracing is of great interest if tracing with incomplete or noisy information is the only possibility. In this regard, we also consider the complex relationship between physical and digital interaction overlap and contact tracing effort. This study can inform health policies aiming to use communication traces for contact tracing.

## Methods

### Contact Tracing

We consider a population of 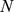 individuals whose connections to each other form a graph. The degree 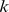 of a node 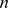 is the number of links between 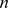 and the other individuals in the population. The mean degree of the network is represented by 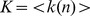 and in random graphs, this degree distribution is Poissonian. Therefore, we do not assume that all nodes have the same degree: the overall node degree distribution forms a Poisson distribution. Individuals can be in one of four different states, susceptible (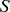), infected (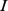), traced (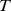), or recovered (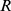). The following state transitions are then defined as in the contact tracing model by Tsimring and Huerta [22], and the symbols are defined in Table 1. Following, we summarize the contact tracing model.

**Table 1:** Dual model symbol description.

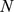	population size
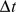	a small interval of time.
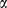	constant determining infection rate.
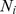	the ideal network in which the epidemic is actually spreading.
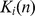	the number of infected neighbours of node 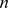 in network 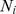 .
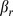	constant determining random tracing rate.
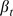	constant determining contact tracing rate.
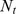	the dual network which is used for contact tracing.
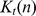	the number of traced neighbours of node 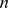 in network 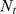 .
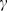	tracing-policy constant controlling the fading time for contact tracing.

#### **Infection** 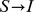


Initially, the whole population is susceptible to infection. One node is subsequently randomly infected, which then starts to infect its neighbours and may initiate an outbreak. The probability that a susceptible node becomes infected is given by 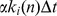, where 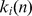 represents the number of infectious neighbours of node n and 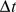 is a small time interval.

#### **Tracing** 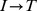


We assume there is no spontaneous recovery, and individuals becomes traced for a certain period of time after which recovery takes place and the individual becomes removed. There are two types of tracing efforts to identify infected individuals, random checking and contact tracing. Random checking is done by choosing an arbitrary node with probability 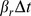. Contact tracing of a node 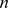 is done with probability 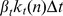, where 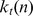 is the number of neighbours of 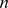 which are in the traced state 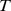. For simplification, traced people can no longer infect other nodes. Additionally, if an infectious individual does not become traced, in the current model it remains infected. An infected node cannot recover (or become removed) unless it is traced.

#### **Removal** 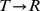


Traced individuals are transformed into the removed state, or recovered state, and are unable to become infected again. A node can recover from the traced state with a probability given by 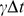.

The contact tracing model can be summarized by the following equations.
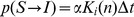
(1)

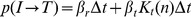
(2)

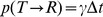
(3)


### Dual Model for Contact Tracing

We first study a dual network topology which accounts for differing edge formations between the infection and tracing networks. Given the contact tracing model defined by equations 1–3, the underlying assumption traditionally is that 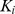 and 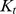 are obtained from one network [22]. Here, we propose to extend this assumption to introduce two networks, one in which disease is spreading, used in equation 1, and one in which tracing occurs, used in equation 2. Our proposed extension, which we refer to as the dual model since it considers two differing networks, represents a more realistic situation in practice which we simulate and validate using a concrete data source from mobile sensed interactions.

We define the network of physical interactions as 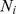. This is the network in which infection transmission takes place. It is also the network from which the number of infected nodes in proximity with node 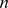, 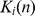, is obtained. In this paper, we assume that this network can never be fully recovered due to, for example, people forgetting whom they interacted with, contagion through strangers and objects, or the impossibility of ever obtaining a complete network of face to face interactions of a population at large [11]. We therefore assume the existence of a dual network, 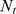, which is a noisy subset of 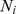. The derivation of 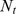 for simulation purposes is described in section “Dual Network Topology”. We assume 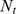 can be obtained by concrete means since it is the network from which tracing will take place. We consider a novel and automatic means of obtaining 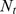 in this paper, by considering 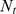 to be taken from ubiquitously sensed interactions obtained by people’s mobile phones. The parameter 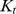 is taken from the dual network 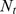, which differs from 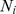 (in [22] 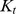 is taken from the same network as 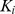).

Next, we propose a formal method for obtaining 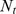 from 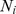, and later demonstrate the differences in simulating such a dual network topology in comparison to an ideal topology. We then demonstrate the successful use of contact tracing on the dual network based on phone call history by simulating the dual network topology over real interaction and communication patterns obtained by a community’s mobile phone logs.

### Dual Network Topology

We define below the process by which we generate the dual network from the ideal network. By removing a portion of the actual ties we simulate a scenario in which the communication traces are only capturing a subset of the actual links. By adding new ties, we simulate the case where communication traces provide dyadic interactions that do not happen in the real world, only in the digital realm. One important measure for our study is the overlap between the two networks, which corresponds to the proportion of links that are present in both networks. The dual network topology is generated as follows:

1. First generate the physical proximity network, 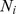, in which disease is spreading.
  - Generate the proximity network of 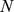 nodes. We assume 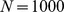 in all of our *simulated* experiments.
  - Generate N*K unique links, where 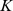 is the average outgoing node degree. This results in 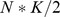 bi-directional links, i.e. the disease can propagate in both directions.
2. Second, generate the dual network, 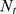, which is a noisy version of the physical proximity network
  - Remove 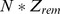 links (and thus 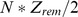 bi-directional links) from the network, where 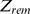 is the average number of forgotten links (per node) which cannot be traced for some arbitrary reason.
  - Add 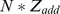 new links which were not among the original ones.

**Figure 1:**
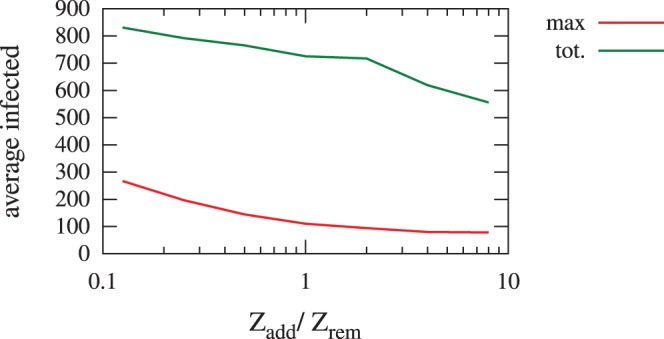
Average maximum and total infected over 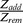. Average maximum and total number of infected people for a network overlap 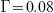, while varying the ratio 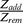 between the number of removed and added edges. The known network (used for contact tracing) is supposed to be a noisy version of the real network (in which epidemics spread), obtained by removing some edges and adding new ones.

In the reported experiments we used 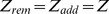. The first motivation to having 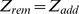 is to be able to vary the overlap between networks while keeping a constant size (number of links) for the tracing network. We thus do not need to apply a corrective factor the 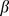 parameters. The second motivation comes from the observation that in real applications, 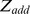 can be either greater than 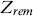 (e.g., if the contact tracing uses a highly connected online social network) or less than 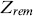 (e.g., if we use meeting agendas of people). Even if we use 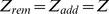, we provide in figure 1 an example of the impact of varying 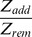 in the case of an overlap of 8%.

Note that 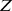 is a noise factor parameter used to generate the dual network. This parameter 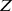 is related to the percentage of overlap, 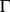, between 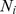 and 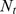 using the following relation:
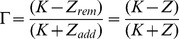
(4)


**Figure 2:**
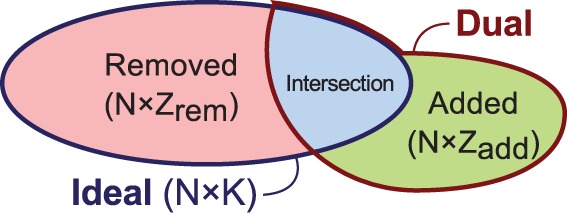
Overlap illustration. Illustration of the overlap in terms of links between the ideal network and the dual network depending on 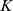, 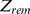 and 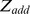. The intersection of the two networks, in blue, is of size 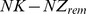 and the union is of size 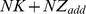.

These equations can be re-derived with the help of figure 2.

### Dataset

We present the dataset that motivates our dual model, and whose parameters, network structure, and dynamics is used in the rest of this paper. The participants in the study represent 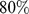 of the total population of an undergraduate dormitory in a North American university previously explored for other applications [23]. The dataset consists of the mobile phone logs of 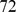 participants, roughly equally distributed across all four academic years, and 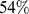 male. The participants used Windows Mobile devices for data collection as their primary phones with their existing voice plans. Data was collected over a 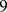 month period between October 2008– June 2009. The data used in this study consists of 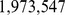 Bluetooth interactions, 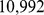 phone call records, and 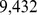 SMS records. The study had IRB approval by the Massachusetts Institute of Technology Committee on the Use of Humans as Experimental Subjects (MIT COUHES) and written informed consent from participants; further details of the dataset can be found here [23].

We consider interaction data logged by the mobile phones. Bluetooth sensors monitored the physical proximity interaction. Other non-physical interactions were monitored by phone communication logs including phone calls and SMS activity. We only consider phone communication and proximity interaction with other study participants (known devices to the study). The data has been previously studied in the framework of real-life health and obesity diffusion [23], [24], opinion diffusion [25], as well as community relationship and interaction pattern analysis [26].

**Figure 3:**
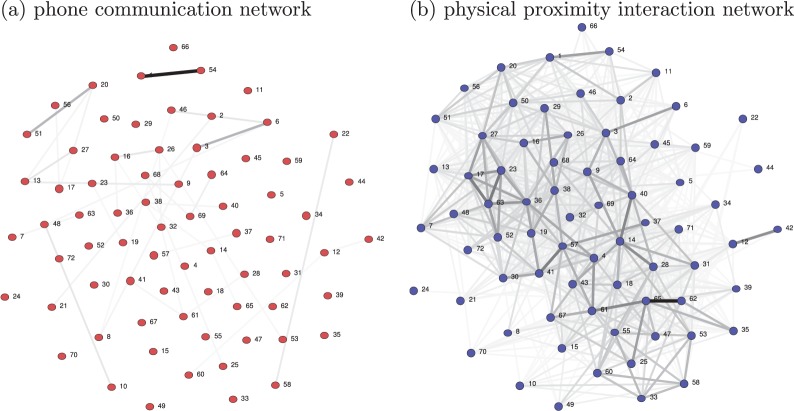
Static network visualizations of the data. The static networks obtained by the overall average number of daily mobile phone (a) communication (call and sms) and (b) physical proximity interactions.

For each of the mobile phone proximity interaction (Bluetooth) and communication (call and SMS) events sensed, we consider the number of events (regardless of their duration), including missed calls. Users correspond to nodes, and undirected edges to interactions. The edges are weighed by the number of events. By considering the number of events, we can readily combine the two types of phone communication logs (calls and SMS). By considering undirected interactions, the proximity interactions can be easily compared to the communication data since phone communication is directed but Bluetooth is undirected. The data is therefore symmetrized, and we assume undirected links. The static average daily networks for the phone communication and physical proximity interactions can be seen in figure 3 (a) and (b), respectively. Note, the nodes are consistent between the two visualizations and the physical proximity represents a highly connected network in comparison to the phone communication network. In this paper (in section “Contact tracing with empirical data in a dual network configuration”), we show that simulating the epidemic spread on network (b) though including its dynamic nature and tracing only based on network (a) suffices in reducing the effects of an epidemic, even given its sparse nature in this dataset.

**Figure 4:**
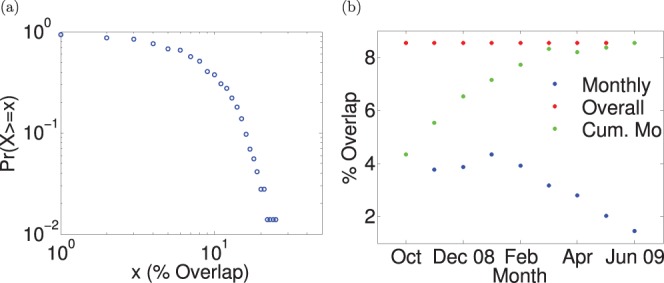
Real data-driven network overlap. (a) Distribution of % overlap between the overall communication and Bluetooth networks on a log-log scale. (b) Monthly variations in the % overlap between the communication and Bluetooth networks averaged over all users.

Next, we consider the overlap between the real physical proximity and communication networks (figure 3 (a) and (b)) in more detail. We obtain the percentage of overlap between the communication network, 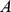, and the physical proximity network, 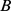, in figure 4, representing a key parameter for our dual model. For each participant, we compute the percentage of overlap within their community, which can be found using the relation 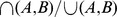. We plot the distribution of the average user’s overlap on a log-log scale. In figure 4 (a), the overlap is considered over an accumulated static network over the entire duration of the study, whereas in (b) the monthly accumulated static networks are considered. First we consider the overall networks. The maximum overlap is 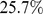, meaning one user communicates by phone with about a quarter of the people they interacted with in the community over the duration of the study. There is never more than 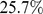 overlap between these networks. The minimum is no overlap (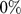), meaning some users never called anyone within the community of people they interacted with. The overall network overlap (in terms of common edges) between the mobile phone network and the Bluetooth interaction network is 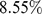. We consider this overlap between the two networks obtained by mobile phone sensed data as an approximate realistic measure for 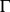, where we set 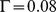 as a lower bound of 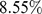 to account for additional measurement error. We can see from figure 4 (a), that the probability of having less than 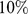 overlap is quite high, and drops significantly for greater than 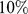 overlap. We do not assume 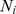 to be equivalent to 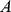, however, as 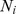 can never be fully obtained in reality, we use the mobile phone data to obtain an estimate of 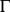. Our experimental results are presented over a range of 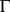, though 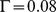 is used as a data-driven approximation and is the focus for the discussion.

In figure 4 (b) we plot the monthly variations in the overlap between the phone and Bluetooth networks. We plot three curves: (1) ‘overall’ is the mean overall average overlap between 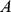 and 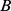 computed over the 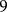 months from (a), (2) ‘monthly’ is only considering the interactions which occurred over the specified month, and (3) ‘cum mo’ is the cumulative monthly, and is all the interactions which occurred up until the specified month. We can see in a specific month, the overlap between these networks is much lower than the average. With the data we are using, we can see that the cumulative monthly approaches the overall monthly after about 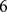 months. This indicates that with our data we approach the mean after about 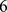 months.

## Simulation Results

### Dual Networks

First we simulate the various network configurations to compare the spread of infection characteristics over the full range of the overlap parameter 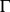. For simulation, we assume a population of 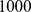 nodes and plot results as averages over 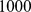 random trials. The model parameters are as follows, 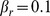, 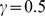, and 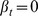 to 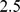. We assume 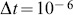. This corresponds to 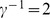 days, 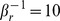 days, and 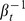 ranging over 10 to 0 days and 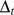 is chosen to be less than 1 second. These parameters correspond to a familiar SIR-type model with rescaled parameters and similar dynamics as in [22]. For comparison, the realistic infectious periods for various infectious diseases can be found in Table 5.1 in [27]. The optimal network case (considered in [22]) occurs at 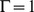, representing 100% overlap between the infectious and tracing networks. The minimal network overlap, 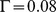, suggested by real data is also highlighted in the results. For every simulation, there is one initial randomly selected infectious case. In the theoretical simulations (not using the real data), we assume 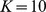.

#### Impact of tracing and network overlap on the size of the outbreak

**Figure 5:**
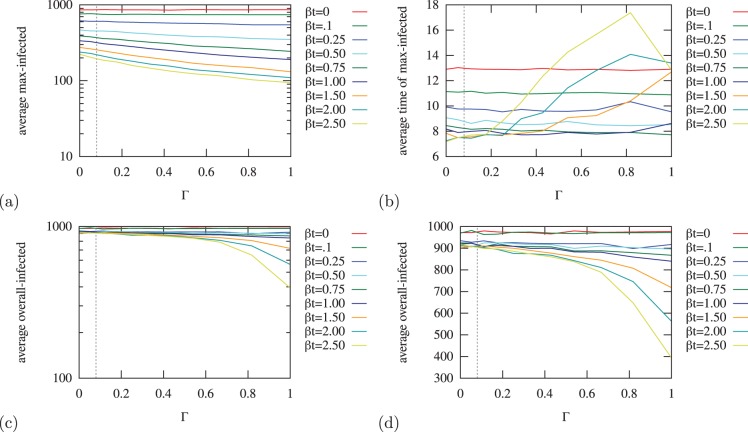
Theoretical epidemic simulations over varying tracing efforts and network overlap. (a) The maximum number of infected individuals (representing the peak of the epidemic), (b) its time of occurrence, and (c)-(d) the overall number of infected individuals on log and non-log scales, respectively; all plotted as a function of 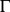, with 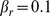 and 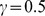. The legend shows the range of contact tracing effort, with 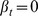 to 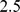. We can see in (a) that contact tracing is effective in reducing the peak number of infected people with 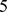 to 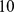 times fewer maximum infected cases between 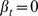 and 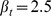. We plot a line at 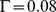, representing a minimal network overlap which corresponds to the values suggested by the analysis of mobile phone data (see figure 4). The greater the overlap between the tracing and disease spreading networks, the more effective the tracing. At the ideal but unrealistic case of 100% overlap, a 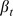 of 2.5 allows to get 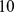 times fewer maximum infected people in comparison to the case with 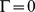. A low overlap such as 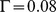 has little effect on the size of the outbreak (the overall number of infected individuals does not decrease much), but still the peak number of infected cases is lowered. With higher overlap, the peak of infections not only decreases in intensity but also gets delayed (c).

**Figure 6:**
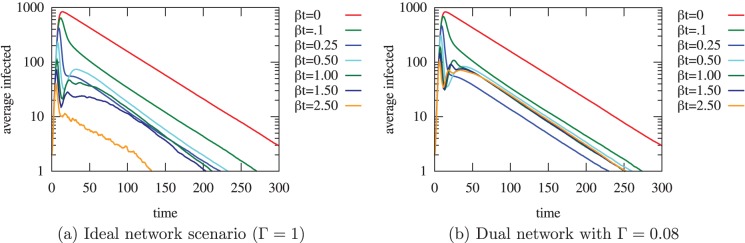
Time varying simulation results of the ideal network scenario and the proposed dual network topology. The infected population plot as a function of time for (a) 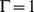 and (b) a network overlap of 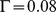, where 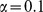, 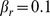, 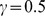, 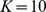. Contact tracing is always beneficial, even when there is a small overlap between 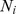 and 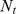. We observe that contact tracing becomes increasingly effective as the number of infections increases in both network topologies (a) and (b). However, contact tracing becomes decreasingly effective as the number of infections decreases, particularly in the dual network topology case. This can be seen by the worsened effects of the second and sometimes third peaks for the dual network case (e.g., with 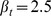).

Figure 5 presents how the peak of the epidemic is affected by the overlap 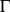, with 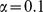, 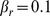, 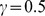, 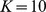. We are considering in figure 5 (a) the value of the peak of the epidemic (i.e., the maximum value in curves such as in figure 6). In general, the greater the overlap between the tracing and disease spreading networks, the more effective contact tracing is. More precisely, the maximum number of infected people decays exponentially with the network overlap (linear slope in a log scale). The intensity of decay increasing with the contact tracing rate 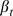.

However, plots from figures 5 (c) and 5 (d) suggest that the total number of infected people behaves differently from the peak value of the epidemic. With a small network overlap (e.g., 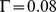), an increased contact tracing rate only slightly changes the total number of infected people. It is only with higher overlap (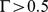) that the contact tracing rate becomes a key factor in reducing the total size of the epidemic.

We are showing, in figure 5 (b), the time at which the peak of the epidemic occurs. This peak occurs earlier when the contact tracing rate is increased. This is due to the fact that the peak is smaller and thus reached earlier. When the tracing rate is high (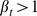), we observe that an increased overlap tends to delay the occurrence of the peak. This reflects the ability of contact tracing (with strong overlap) to effectively slow down the spread of the disease.

#### Temporal impact of contact tracing

**Figure 7:**
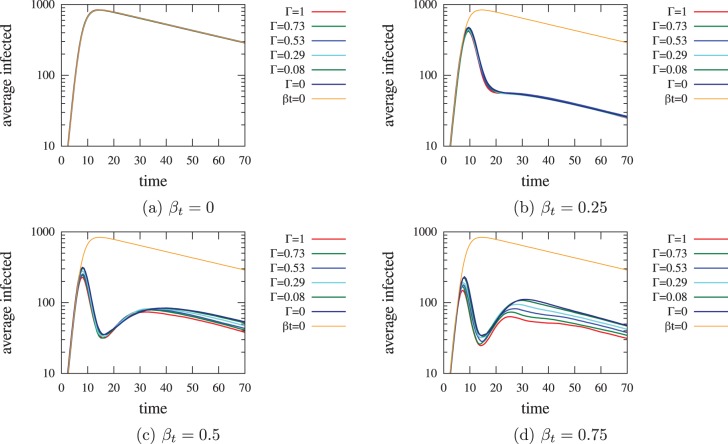
Time varying simulation results of our proposed contact tracing dual network topology while varying network overlap, 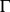. We observe the changing effects of the time-varying spread over 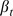. The difference in infectious spread over time becomes more apparent in the cases with two peaks, where 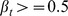 particularly after the second peak, where an increase in network overlap results in fewer infected cases. Note, the log scale employed to make the graphs easily comparable tends to attenuate the differences between curves within a graph.

The time-varying nature of the epidemic can be seen in figures 6 and 7 where the log of the average number of infected individuals is plot over time. Figure 6 shows the case with full overlap (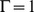) in comparison to the case with 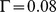 overlap, which is the lower bound on the actual average network overlap found in the real social interaction networks (see figure 4). We can see that even with such a low percentage of network overlap, contact tracing continues to be effective as it notably reduces the outbreak of the epidemic. In general, we observe that contact tracing becomes increasingly effective as the number of infected cases increases. In many of the cases there are two to three peaks in the epidemic and the number of infected individuals over time. The differences between the two network configurations 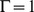 (figure 6 (a)) and 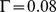 (figure 6 (b)) becomes increasingly apparent as the amount of tracing increases whereby in reality, the more realistic network configuration (b) shows even a decreased amount of tracing 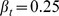 may result in fewer cases and a more optimal tracing scenario. This occurs due to the fact that there is no second epidemic peak in this scenario (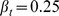), though with larger tracing effort, the epidemic is reduced significantly rapidly, leaving a great deal of the population susceptible for a second peak of infections.

In figure 7 we consider four cases of fixed 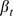 to observe the difference in the effects of the percentage of network overlap in contact tracing. Again, 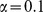, 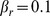, 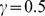, 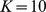. We see the worst case occurs for no contact tracing based on social interactions (plot (a) where 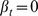). Different amounts of contact tracing have differing time-varying effects on the disease spreading. For small amounts of contact tracing there is only one peak ((b) 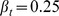). However as soon as 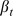 increases beyond that point, there are two peaks ((c) 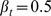, (d) 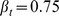). This is due to the contact tracing becoming so effective that the number of cases drops rapidly, resulting in tracing becoming less effective. Note, this effect is much more attenuated in the dual network case (figure 6 (a) versus (b)) which is the scenario closer to reality due to incomplete network information. This is due to having a smaller probability of tracing effectively (i.e. tracing an infected contact) given the smaller number of infectious cases, whereas in an optimal network case there is a higher chance of effective tracing given a smaller number of infections.

#### Why does contact tracing work with such low overlap?

Our results have shown that even with very small overlap between the two networks, contact tracing was still effective in limiting the peak size of the epidemic. With low overlap this behaviour might be surprising. It is actually explained by a simple fact: when using contact tracing, an increase in the number of infected people causes an increase in the tracing effort. This adaptation phenomenon is not present when only random tracing is used. We aim here at quantifying whether it is still worth doing contact tracing with a relatively small network overlap or if increasing random tracing is preferable.

We measure the tracing effort defined as the sum of the effort due to random tracing and the effort due to contact tracing:
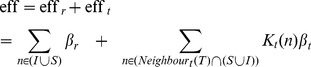
(5)


What the formula encodes is that the random tracing (with intensity 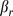) is done on the population of both infected and susceptible nodes, as we cannot know in advance who is infected when doing tracing. Similarly, the effort due to contact tracing (with intensity 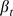) targets a population made of all the neighbours (in the tracing network) of traced nodes that are still either susceptible or infected.

**Figure 8:**
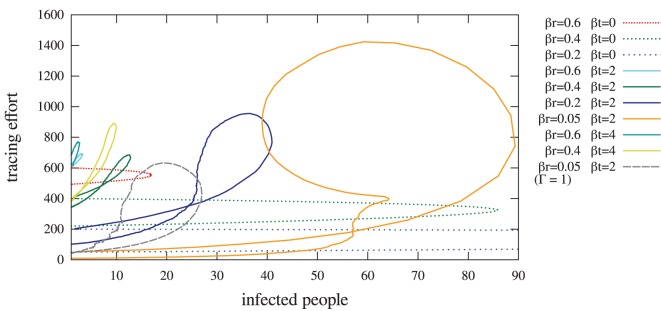
Average temporal evolution of the tracing effort and the number of infected people with or without contact tracing. Only the last curve considers the case with complete network overlap (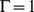) while all other curves are with 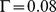.

In figure 8, we plot both the number of infected nodes and the total tracing effort. Results are presented for the low network overlap case, 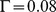, unless specified otherwise. The three dotted curves represent the tracing effort profile when no contact tracing is used. Considering the dotted blue curve with only random tracing, 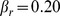 and 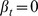, the tracing effort starts at 200 (1000 individuals times 0.20), and the number of infected nodes grows up to 300 (not shown), then it decreases greatly at the end of the epidemic. Generally, the tracing effort is smaller at the end of the epidemic because the traceable (susceptible and infected) population is reduced. Now, with the addition of contact tracing (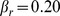 and 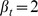), the tracing effort increases greatly as the epidemic grows, however there is a significant reduction in the number of infected cases (below 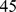 cases as opposed to 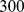).

Comparing the solid green curve (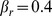, 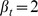) with the dotted red curve (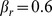, 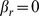), figure 8 results show that it can preferable to design a tracing policy that employs a lower random tracing rate but that uses contact tracing (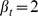, even with an overlap of 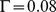). There is a trade-off to consider in terms of tracing effort over time, maximum tracing effort, and peak in number of infected individuals. While at the peak of the epidemic, the tracing effort does increase beyond the case with random tracing alone, this increase in tracing effort is not constant. The effort is less than for the random tracing effort alone at the start and end of the epidemic. Further, the peak of the epidemic is reduced when considering the addition of contact tracing with reduced random tracing (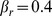, 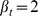 vs. 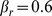, 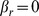). A similar trend is observed for varying the level of random tracing, and considering a reduced random tracing with the inclusion of contact tracing. In figure 8, the case of 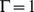 illustrates the positive impact of having perfect knowledge of the spreading network. We observe that better knowledge of the network highly reduces the size of the outbreak down to 26, while allowing for very low random tracing (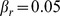 instead of, e.g., 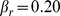).

In general, contact tracing does not require a great effort at the beginning of the outbreak, but rapidly becomes costly when the epidemic evolves. However, it is effective in reducing the size of an epidemic with low network overlap, as is random tracing alone. An optimal solution to consider in future work may be to consider varying the random and contact tracing efforts over time to optimize costs as the epidemic evolves. A tracing policy including contact tracing allows to both adapt tracing to the number of infected people and exploit the known information about people’s interaction. Such policies have the potential of reducing the constant efforts required by random tracing and considering the use of contact tracing at particular intervals while containing an epidemic outbreak with minimal cost.

#### Relative proportion of contact tracing and random tracing

We observed that one benefit of contact tracing over pure random tracing is that it adapts the tracing effort to the number of detected infections and thus has a varying effort (and cost) over time. To further explore the role of contact tracing, we consider a setup where we assume a fixed amount of tracing effort is available. In such a case, we expect and observe that contact tracing with a low overlap is not advantageous.

In the simulation, we allow a fixed tracing budget (400 units). We allocate a fixed part of this budget to random tracing, the rest goes to contact tracing. In practice, we continuously adapt the 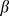 parameters to spread the tracing budget between contact tracing and random tracing, in the desired proportions. A special case happens at instants when no individual is in state T (traced after being infected), mostly at the beginning of the epidemic. In this case, the full budget goes to random tracing.

**Figure 9:**
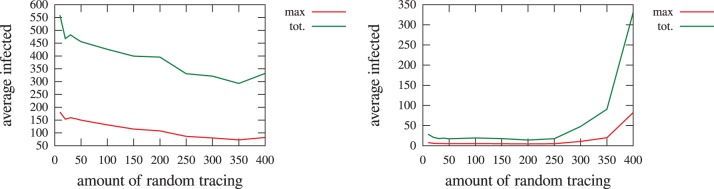
Average maximum and total numbers of infected people against the amount of random tracing effort. Simulations consider a network overlap of 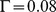 (left) and 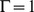 (right), when the total tracing effort is constant (400).

Figure 9 shows the average maximum (and total) number of infected people depending on the part of the budget dedicated to random tracing. We provide curves with two different network overlaps (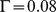 (left), 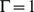
(right)). With complete overlap, we clearly observe that contact tracing is strongly beneficial. Even with this perfect knowledge of the network, results show that spending more than half of the budget in contact tracing is not providing improvement (nor degradation). With low overlap (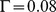), simulation results show that doing some contact tracing is better than not doing any but that it soon becomes detrimental. From these experiments we can conclude that, with low overlap, the biggest cause of the previously observed positive impact of contact tracing is actually the adaptive tracing effort.

### Contact Tracing with Empirical Data in a Dual Network Configuration

**Figure 10:**
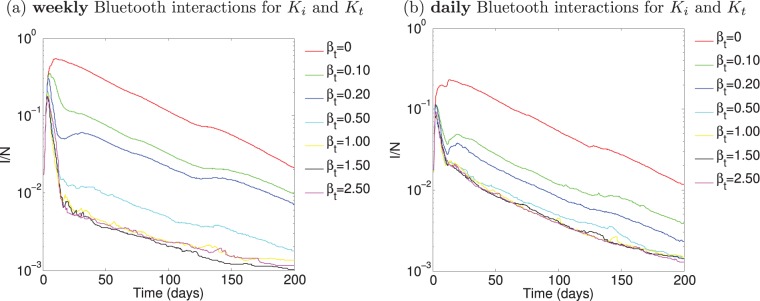
Simulation of contact tracing over the empirical data with 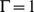. Only the real physical proximity interactions are used to obtain 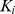 and 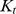. The physical proximity interactions are obtained by the mobile phone Bluetooth data and are incorporated on (a) a weekly scale, and (b) a daily scales.

**Figure 11:**
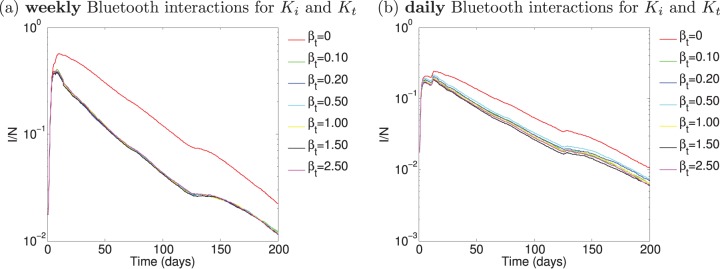
Dual network scenario simulated over the real mobile phone data. Bluetooth physical proximity is used for 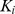, phone communication logs are used for tracing, 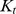.

In this section, we consider experiments on the real data. We apply the dual contact tracing model on the full empirical interaction and communication dataset obtained by mobile sensing. While the physical interactions obtained by Bluetooth are not a complete picture of the interaction history, they do represent a large portion of the interactions (subjects were explicitly asked to leave their Bluetooth on all the time). We consider two timescales over which the real interaction data is aggregated, daily and weekly. The two timescales are chosen to consider the time-specific nature of real data in our evaluation and to simulate the dynamics from real data considering two easily interpretable timescales. The results referred to as practice (as opposed to theory), are simulated only considering the empirical data (daily and weekly); the real interaction events occurring within the community are used to model the dynamics of the parameters 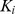 and 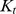. The interactions obtained by the Bluetooth physical proximity are aggregated over weekly and daily intervals and used directly for 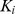 to simulate the epidemic. Similarly, the interactions obtained by Bluetooth are first used directly to define the tracing effort (i.e. to determine 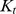) on weekly and daily timescales in figure 10, considering the case where 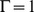. Next, the tracing effort (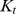) is only determined by interactions occurring in the phone communication network to determine the effects of tracing from the phone communication network in figure 11. In this case 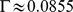, which is the real overlap between these networks inherent to the dataset. The interactions are weighed according to the following equations. 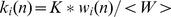, where 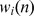 represents the total number of interaction events between node 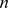 and its infectious neighbours within the specified time frame and 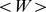 is the average weight over all connected participants. 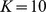 in all simulation results presented. Overall, 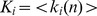 (in equation 1) is the mean degree of the infectious interaction network, which is a reweighing of 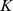 (used in the theoretical simulations) according to the real data. Similarly, 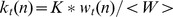.

**Figure 12:**
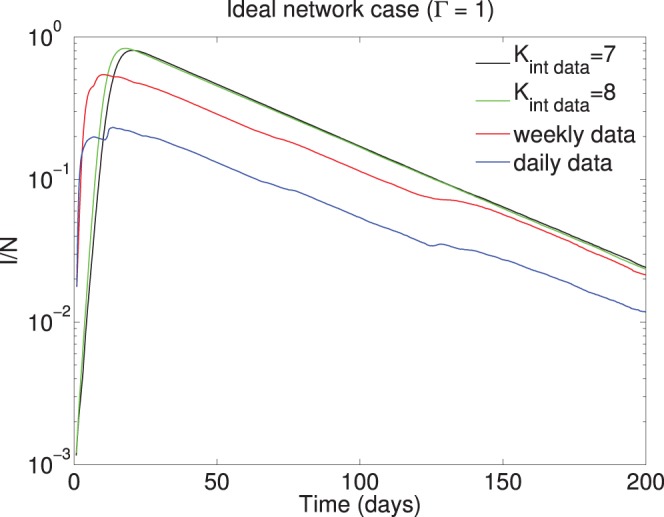
Theory versus practice. Considering the ideal network scenario, we run the simulated contact tracing model with 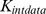 set to the average daily (and weekly) node degree of the data (see figure S1), but consider a simulated network (labeled as 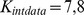). Two data-driven models are considered with the interactions taken from the Bluetooth proximity logs. For all cases, 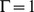, and therefore 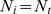. The real data is considered on weekly and daily scales, and 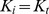 are the real physical interactions logged by the community’s Bluetooth sensors.

First, we evaluate the difference between using the physical proximity data in an ideal network scenario in comparison to the theoretical case by comparing the model outputs on this community of 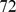 participants (note 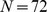 in this section). The contact tracing model results obtained over the data-driven proximity network are labeled as weekly data and daily data in figure 12. For the case 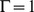, the interactions for 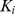 and 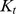 are both taken from the physical proximity data since we are assuming perfect network overlap. We assume 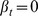 to remove the additional effects of tracing in the comparison. What we refer to as the theoretical case is the contact tracing model simulated according to equations (1)-(3) without any real data for 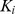 and 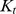. For the theoretical case, the mean degree of the physical proximity network is used to set the network weights; 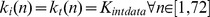, where 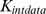 is the overall node degree of the mobile phone proximity network (refer to figure S1). Figure 12 shows the difference between the theoretical case and the contact tracing model run over the physical proximity network. We observe the results considering the weekly aggregated interactions are in close agreement with the theoretical case. This confirms the Bluetooth interaction data collected for this community considered on a weekly scale to be in good agreement with the theoretical model, though not equivalent. The incorporation of daily interaction data results in almost an order of magnitude difference in the peak of the epidemic illustrating the models generally over estimate in theory due to less sparse weekly interaction patterns in reality. Precise details of the simulation scenario can be found in “Simulation Details” in File S1.

After making a comparison of the theoretical case with the data-driven case, whereby only the physical proximity network is considered in simulating infectious spread, we evaluate the proposed dual network methodology entirely on the real dataset. First we consider the single network case (with 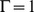) in figure 10. Again, the real physical proximity interaction network is the underlying network both for infection spread and contact tracing, considering (a) weekly aggregated interactions and (b) daily aggregated interactions. In these simulations, we are evaluating the effect of tracing on Bluetooth physical proximity data which could potentially be available for tracing by service providers. We observe that contact tracing is very effective in reducing the effects of the epidemic both on a weekly and daily rate. In figure 10 (a) we observe an optimal tracing strategy occurs on this dataset for 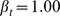, which demonstrates maximizing tracing efforts is not always the best strategy, tracing efficiently is more important. There is no significant difference in the number of infected cases over time when 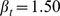 or 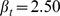, meaning this additional effort is wasted in comparison to 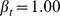. The number of interactions occurring on a daily scale diminishes in comparison to the weekly case, and therefore the tracing effort reaches its lower bound sooner (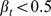). However, even on such a fine-grained timescale of one day, contact tracing based on the Bluetooth physical interactions captured by the mobile phone plays a significant role in reducing the effects of the epidemic. Next, we consider tracing based on only the phone calling records obtained in the data collection.

In figure 11 we consider the dual network case on the mobile phone data, where the infection spreads on the physical proximity network, and the tracing occurs only on the phone communication network. In figure 11 (a) the weekly aggregated physical proximity and phone communication networks are considered and in (b) the overall aggregated daily networks are considered. While we demonstrate in figure 4 the overall average overlap between these networks is 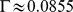, we see the effect of the epidemic is greatly reduced by tracing using the phone call records of the mobile phone users. These effects are less visible on the daily scale than the weekly scale which is due to the small number of daily phone communication traces made in the dataset (see figure S1 (a)). The results of tracing on the phone communication records illustrate that a very small tracing effort (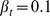) is sufficient in reducing the effects of the epidemic and is further a concrete and easily obtainable source for tracing. Note, the participants in this community are more likely to make fewer calls to one another since they are living together in a dormitory. However even given this challenging data collection, the results are still very significant, particularly on a weekly scale, whereby tracing based on users’ phone call records over a given week results in the reduction in the effects of an epidemic outbreak.

## Discussion

We explore a data-driven avenue for contact tracing in epidemic prevention using social interaction data from mobile phones. A medium-sized real communitys data is considered to get insight into the relationship between physical interactions and mobile phone communication, and whether the latter can be exploited to perform contact tracing on the former. We explore the effectivity of such a strategy using data-driven simulations with realistic parameters extracted from the social network dataset, first, and then the full dual realistic network model of physical and communication interactions. Across multiple realistic scenarios for contact tracing, we find that contact tracing is an effective means for epidemic prevention, even when there exists a low overlap between the physical and communication networks. When considering tracing effort, we observe that contact tracing is greatly beneficial when the epidemic is starting, however, this effort will increase greatly as the epidemic grows. With low overlap between the physical and communication networks, we find that this effect is mainly due to the automatic adaptation of the tracing effort to the amount of infected people. We also uncover the relationship between the network overlap and the proportion of effort spent in random tracing versus contact tracing. The study thus gives insight into what proportion of the effort should be spent in contact tracing depending on the estimated network overlap (how much we trust the communication network represents the interaction network). While contact tracing is effective in reducing the number of infected cases, a dynamic approach considering a time-evolving combination of random and contact tracing is most promising, and optimization of costs as a function of varying random and contact tracing efforts over time will be considered in future work. We are also able to uncover the nonlinear relationship between overlap (between physical and communication networks) and contact tracing effort. This is important, as different communication technologies, present and future, are likely to have a different link to physical interactions. Quantifying how the overlap interacts with the tracing effort can inform public health policies aiming to exploit digital communication traces for epidemiology. Overall, we find interactions sensed by mobile phones to be a promising tool for epidemic simulation, particularly for future large-scale scenarios, for example city-scale infectious disease transmission. This work demonstrates mobile phone communication history to be a useful data source in disease prevention by obtaining contact information readily for epidemic contact tracing.

## Supporting Information

Figure S1 **Averaged user node degree per individual days (or weeks) in the study.** These node degree distributions are plot on a daily basis (a)–(b) and on a weekly basis (c)–(d). The node degrees averaged over the users and over the days (or weeks) are used to simulate the epidemic in figure 12.
(EPS)

Figure S2 **Number of events logged over time.** We observe that SMS activity is equally important to consider as call activity in mobile phone communication data. Bluetooth interaction data is highly dependent on time, more so than the phone activity, which remains more constant over time.
(EPS)

Figure S3 **Community’s overall network structure.** The average node degree as a function of the minimum edge weight for (a) the communication network (call and SMS) and (b) the physical proximity interaction network. Edge weight is defined as the minimum number of events necessary for an edge to connect two nodes in both networks indicating the tie strength between the pair of nodes. The units in both cases are the number of events.
(EPS)

Figure S4 **Node degree distributions.** Accumulated node degree distribution over the study duration for (a) the phone network and (b) the physical proximity interaction network. Each individual user’s node degree is obtained as an accumulation over the 9 month period, and the distribution is plot given one overall node degree per user. The node degree for the accumulated static phone network is 7∶3 and for the Bluetooth network is 67∶25.
(EPS)

File S1 (PDF)
