# Supplementary material for: Epidemic Contact Tracing via Communication Traces
Source: PLoS One. 2014 May 1;9(5):e95133. doi: 10.1371/journal.pone.0095133 (PMC4006791; doi:10.1371/journal.pone.0095133)
Supplement: File S1 — (PDF) [file pone.0095133.s005.pdf]

Supporting Information

Mobile Phone Data

The number of daily events for the phone communication and physical proximity datasets is plot in figure S2. The SMS activity is just as prevalent as phone call activity in this community. The physical proximity activity is dependent on time of year, more so than the phone communication activity which is more constant overall. The drop in physical proximity activity at approximately day 100 coincides with Christmas holidays where students go home and interactions become sparse.

The average node degree is plot as a function of the edge weight on the static network structures in figure S3. The edge weight determines the minimum number of events that need to occur for an edge to connect two nodes indicating the strength of the tie. Looking at figure S3 (a), the phone communication network (phone calls and SMS activities) is not as heavily connected as the physical proximity network in (b). On a log-log scale, the phone communication connectivity drops linearly with the tie strength, however the Bluetooth interaction drops exponentially.

In figure S4, the node degree distributions are plot over the accumulated static network data. These results indicate the range of connectivity K_t, which can be obtained from real interaction sensors by mobile phones. The average node degree for the phone communication network (phone + SMS) is 7.3, whereas, for physical proximity in this highly knit community, it is 67.25. Relating these values to our simulated networks, the corresponding parameters are $K = 67.25$, $Z_{add} = 1.8$, $Z_{rem} = 61.7$ and $\frac{Z_{add}}{Z_{rem}} = 3\%$, with $\Gamma = 8\%$. The node degree distributions are additionally considered on the overall accumulated daily networks and weekly networks in figure S1. These results give a more realistic measure for an approximate value of the average number of unique individuals a person will interact with on average, resulting in a data-driven measure of K_i. The average daily K_i is 7.17 for the physical proximity network, and 7.51 for the average weekly measure.

Simulation Details

For computer simulation of the epidemics, we consider a continuous time approach as opposed to a discrete time approach. A continuous time approach entails sampling the next time at which a node will change its state, whereas a discrete time approach samples a new state for each node at every small discrete time step. The discrete time approach is very slow, particularly if there are many edges in the network. For simulations, we consider the three state changes given by equations 1-3. At time t, for each susceptible node n, we consider the number of infected nodes which were in proximity to n given by $k_i(n)$. $k_i(n)$ is obtained as the overall weekly or overall daily physical proximity interactions obtained by the mobile phone Bluetooth data, changing dynamically over the 31 week period when simulations take place over the data. In the cases not simulated over real data, $K_i = K = 10$. When $\Gamma = 1$, K_t is equivalent to K_i since we assume perfect network overlap (for example, in figure 10). When $\Gamma < 1$, we refer to this as the dual network case, and K_t is taken from the weekly or daily overall phone communication interactions (for example in figure 11).

For the data-driven simulations, the node weighings are performed as follows. For each susceptible node, we sample the next time at which a state change will occur, considering equation 1, $k_i(n) = K * w_i(n) / < W >$, $k_t(n) = K * w_t(n) / < W >$, $\alpha = 0.1$, $\Delta t = 10^{-6}$, $\gamma = 0.5$. $w_i(n)$ represents the total network weights between user n and its infected neighbours taken from the real data, and $< W >$ is the overall static average network weight. In the case with perfect network overlap (figure 10), W is taken from the mobile phone physical proximity network for both K_i and K_t. In the dual network case (figure 11), W is taken from the physical proximity network for K_i and from the phone communication network for K_t. Note, in the daily simulations, $w_i(n)$ corresponds to the overall accumulated daily interactions occurring between user n and its infected neighbours. On the weekly scale, $w_i(n)$ corresponds to the overall accumulated weekly interactions between user n and its infected neighbours. Similarly, $w_t(n)$

corresponds to the overall accumulated daily or weekly interactions with traced neighbours of n. We sample the next time at which a state change will occur for every susceptible, infectious, and traced node using equations 1-3, respectively. The single node at which the minimum amount of time is sampled is chosen and that node's state is updated. The procedure of next time sampling and state change updating continues until reaching a maximum point in time, or until all nodes have recovered. By sampling times of state change as opposed to state changes for very small discrete times, the procedure can be simulated much more quickly. We assume one randomly selected infectious case initially, perform 1000 simulations for each curve, and plot the average of the 1000 random trials for each result presented.

Figure S1. Averaged user node degree per individual days (or weeks) in the study. These node degree distributions are plot on a daily basis (a)-(b) and on a weekly basis (c)-(d). The node degrees averaged over the users and over the days (or weeks) are used to simulate the epidemic in figure 12.

Figure S2. Number of events logged over time. We observe that SMS activity is equally important to consider as call activity in mobile phone communication data. Bluetooth interaction data is highly dependent on time, more so than the phone activity, which remains more constant over time.

Figure S3. Community's overall network structure. The average node degree as a function of the minimum edge weight for (a) the communication network (call and SMS) and (b) the physical proximity interaction network. Edge weight is defined as the minimum number of events necessary for an edge to connect two nodes in both networks indicating the tie strength between the pair of nodes. The units in both cases are the number of events.

Figure S4. Node degree distributions. Accumulated node degree distribution over the study duration for (a) the phone network and (b) the physical proximity interaction network. Each individual user's node degree is obtained as an accumulation over the 9 month period, and the distribution is plot given one overall node degree per user. The node degree for the accumulated static phone network is 7.3 and for the Bluetooth network is 67.25.
